# A Three-Stage Colonization Model for the Peopling of the Americas

**DOI:** 10.1371/journal.pone.0001596

**Published:** 2008-02-13

**Authors:** Andrew Kitchen, Michael M. Miyamoto, Connie J. Mulligan

**Affiliations:** 1 Department of Anthropology, University of Florida, Gainesville, Florida, United States of America; 2 Department of Zoology, University of Florida, Gainesville, Florida, United States of America; University of Utah, United States of America

## Abstract

**Background:**

We evaluate the process by which the Americas were originally colonized and propose a three-stage model that integrates current genetic, archaeological, geological, and paleoecological data. Specifically, we analyze mitochondrial and nuclear genetic data by using complementary coalescent models of demographic history and incorporating non-genetic data to enhance the anthropological relevance of the analysis.

**Methodology/Findings:**

Bayesian skyline plots, which provide dynamic representations of population size changes over time, indicate that Amerinds went through two stages of growth ≈40,000 and ≈15,000 years ago separated by a long period of population stability. Isolation-with-migration coalescent analyses, which utilize data from sister populations to estimate a divergence date and founder population sizes, suggest an Amerind population expansion starting ≈15,000 years ago.

**Conclusions/Significance:**

These results support a model for the peopling of the New World in which Amerind ancestors diverged from the Asian gene pool prior to 40,000 years ago and experienced a gradual population expansion as they moved into Beringia. After a long period of little change in population size in greater Beringia, Amerinds rapidly expanded into the Americas ≈15,000 years ago either through an interior ice-free corridor or along the coast. This rapid colonization of the New World was achieved by a founder group with an effective population size of ≈1,000–5,400 individuals. Our model presents a detailed scenario for the timing and scale of the initial migration to the Americas, substantially refines the estimate of New World founders, and provides a unified theory for testing with future datasets and analytic methods.

## Introduction

For decades, intense and interdisciplinary attention has focused on the colonization of the last habitable landmass on the planet-the peopling of the Americas. The first comprehensive, interdisciplinary model for New World colonization incorporated linguistic, paleoanthropological, and genetic data and generated great controversy, which was due at least in part, to the uniquely broad scope of the research [1]. Since that time, more focused studies have resulted in agreement on the general parameters of the colonization process, such as a single migration in contrast to the original three-migration model that distinguished Amerinds, Na-Dene, and Eskimo-Aleuts [1]. However, a full understanding of the complex and dynamic nature of the timing and magnitude of the colonization process remains elusive.

The majority of the genetic literature supports a single migration of Paleoindians into the New World from an East Asian source population [2]. Specifically, the reduced variation and ubiquitous distribution of mitochondrial and Y chromosome haplogroups and microsatellite diversity throughout the New World relative to Asia argue strongly for a single migration [3], [4]. However, a great many models have been proposed that differ significantly in the timing and size of this migration event [2], [5]–[15]. Different migration dates have been proposed ranging from ≈13 thousand years ago (kya) to ≈30–40 kya [2], [5]–[15]. Numerical estimates of the founder effective population size (N_e_) are infrequent in the literature but vary substantially, from a high of ≈5000 [6] to a low of ≈70 Paleoindian founders [16]. These dates and population sizes have been proposed to accommodate a wealth of scenarios including ancient, recent, and/or additional migrations responsible for the peopling of the Americas.

Archaeological data provide clear support for a widespread human presence in the Americas by ≈13 kya (all calendar dates are recalibrated radiocarbon dates as reported in the cited literature), the time by which the Clovis complex was established across the interior of North America [17], [18]. Older archaeological sites, e.g. the Nenana Complex in Alaska [18], the Monte Verde site in Chile [19], and the Schaefer, Hebior and Mud Lake sites in Wisconsin [20], [21], document an earlier chronology possibly 2,400 years before Clovis [18], [20], [21]. Additionally, very old radiocarbon dates have been obtained from sites in Asian Beringia suggesting that human populations had reached the north of western Beringia by ≈30 kya [22], [23].

The geological and paleoecological records for Beringia and northwestern North America provide further constraints on the timing for the peopling of the Americas. Beringia was a continuous landmass that connected Asia and North America roughly 60 kya until ≈11–10 kya [23]–[25]. However, Beringia was isolated from continental North America until ≈14 kya when an intracontinental ice-free corridor opened up between the Laurentide and Cordilleran Ice Sheets [26]. Paleoecological data indicate that Beringia was able to sustain at least small human populations. Fossil pollen and plant macrofossils from ancient eastern Beringia are indicative of a productive, dry grassland ecosystem [27] and paleontological evidence from Alaska and Siberia demonstrates that large mammals roamed Beringia [28].

After 11–10 kya, Late Pleistocene sea levels rose sufficiently to re-inundate Beringia [24], [25], creating the Bering strait that now separates the New World from Siberia by at least 100 kilometers (km) of open frigid water. Studies of human settlement throughout the Pacific Islands indicate that open water distances of >100 km constitute significant barriers to human migration, possibly because ancient people were unlikely to travel further than one day out of sight of land [29]. Similar constraints (if not worse) would apply to early humans in Alaska and Siberia, thereby severely reducing the migration rate between the New and Old World once Beringia was re-inundated. Reduced migration due to the Bering Strait remains valid even as recent rates of short-range migration have increased between Siberia and Alaska [13]. In effect, the two continents were essentially geographically isolated from 11–10 kya until modern times.

No detailed, unified theory of New World colonization currently exists that can account for the breadth and complexity of these interdisciplinary data. We analyze Native American mitochondrial DNA (mtDNA) coding genomes plus non-coding control region sequences as well as a combined nuclear and mitochondrial coding DNA dataset from New World and Asian populations. Mitochondrial DNA data represent the ‘gold standard’ of genetic data types and provide the most extensive comparative database for human populations worldwide [30]. Furthermore, it has been proposed that mtDNA may be more sensitive to demographic changes, such as population bottlenecks, due to its smaller effective population size [31]. The combined nuclear and mtDNA dataset was recently used to propose an unusually small N_e_ for the Amerind founders [16], and thus investigation of this dataset is of much interest when attempting to reconcile the existing genetic evidence. We use two complementary coalescent methods to develop a comprehensive scenario of New World colonization, with a focus on the timing and scale of the migration process. Bayesian skyline plot analyses use data from a single population to provide an unbiased estimate of changes in N_e_ through time, and thus are a powerful means for estimating past population growth patterns when the nature of the growth (e.g. exponential or constant) is unknown [32]. The isolation-by-migration (IM) structured coalescent model uses data from sister populations to jointly estimate population divergence time, migration rates and a founder N_e_, with an assumption of exponential growth [16]. Importantly, we explicitly incorporate archaeological, geological, and paleoecological constraints into both analyses. Our goal is to provide a comprehensive model for the initial settlement of the Americas that generates new testable hypotheses and has high predictive power for the inclusion of new datasets. In light of our results, we propose a three-stage model in which a recent, rapid expansion into the Americas was preceded by a long period of population stability in greater Beringia by the Paleoindian population after divergence and expansion from their ancestral Asian population.

## Results

### Skyline Plot Analyses

**Figure 1:**
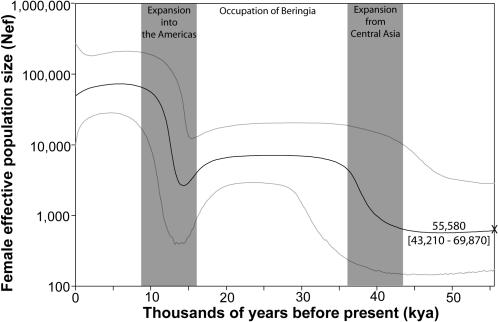
Bayesian skyline plot for the mtDNA coding genome sequences. The curve plots median N_ef_ with its 95% CI indicated by the light gray lines. The calculated N_ef_ assumes a generation time of 20 years following Hey [16]; alternatively, using a generation time of 25 years [55] would uniformly decrease all estimates of N_ef_ by 20%. “X” marks the median coalescent time with its 95% CI given in brackets. The shaded regions highlight two periods of substantial population growth. This skyline plot provides the principal evidence for our three-stage model of New World colonization, i.e. the three stages that are depicted and labeled here.

Our alignment of 77 full mitochondrial coding genomes is one of the largest published alignments of Native American mtDNA coding genomes (Figure S1). It includes genomes from the four major mtDNA haplogroups in the Americas (haplogroups A, B, C, and D are each represented by 17–31% of the entire sample), as well as the minor haplogroup X (2%). Correspondingly, this set of 77 complete coding mtDNA genomes represents geographically and linguistically diverse populations distributed throughout the New World [3]. Bayesian skyline plots [32] were used to visually illustrate changes in Amerind female effective population size (N_ef_) over time. Bayesian skyline plots assume a single migration event, which makes the approach ideal for questions concerning the peopling of the Americas since it is generally agreed that there was a single migration [3]. Our skyline plot of the coding genomes describes a three-stage process in which there are two distinct increases in N_ef_ at ≈40 kya and ≈15 kya that are separated by a long period of little to no growth (Figure 1). Specifically, N_ef_ increases from ≈640 [95% credible interval (CI) = 148–9,969] to ≈4,400 individuals (95% CI = 235–18,708) at the first inflection point, and from ≈4,000 (95% CI = 911–13,006) to ≈64,000 individuals (95% CI = 15,871–202,990) at the second inflection point. There is also an apparent decrease in N_ef_ prior to the second inflection point in which median N_ef_ drops to ≈2700 (95% CI = 404–36,628). We define a significant change in population size as the occurrence of non-overlapping 95% CIs at the beginning and end of an increase (see shading in Figure 1). Thus, we interpret the recent ≈16-fold increase in N_ef_ over the interval ≈16–9 kya as significant. The earlier ≈7-fold increase at ≈43–36 kya is suggestive but not significant, although the increase is significant when compared over a much longer time period, e.g. from ≈25 kya to the coalescent. Overall, the recent increase is consistent with a rapid, large-scale expansion into the Americas while the older increase is suggestive of a gradual expansion within Asia or Beringia.

**Figure 2:**
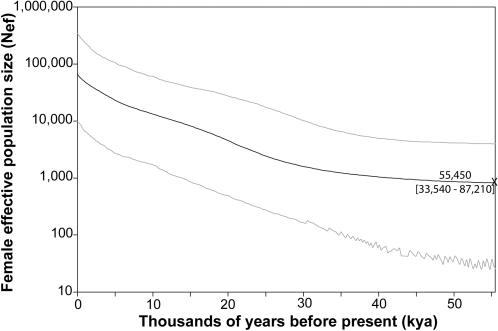
Bayesian skyline plot for the mtDNA HVR I+II datasets. This plot follows the conventions of Figure 1. Its estimates of coalescent time and N_ef_ at the coalescence and today are in agreement with the coding mtDNA skyline plot (Figure 1). In contrast, this HVRI+II plot provides little resolution for other population size changes, most likely because of mutational saturation in the non-coding control region (see text).

The dataset of 812 concatenated mtDNA hypervariable region (HVR) I and II sequences is one of the largest published alignments of Native American HVRI+II sequences (Figure S2). It includes all major New World haplogroups, and represents geographically and linguistically diverse populations distributed throughout the Americas. The HVRI+II dataset was randomly divided into ten non-overlapping alignments of 81 HVRI+II sequences, which allowed for ten independent trials for parameter estimation with a sample size similar to the coding genome alignment. The HVRI+II skyline plot analyses (Figure 2) produce estimates for median time to coalescence (55.5 kya, 95% CI = 33.5–87.2 kya) and N_ef_ at coalescence (820, 95% CI = 26–3,979) and the present (66,200, 95% CI = 9,839–346,289) that are similar to the coding genome analyses (Figure 1). However, in contrast to the coding genome skyline plot, the HVRI+II skyline plot traces a very gradual increase in N_ef_ over ≈40,000 years with no clear inflection points. The HVRI+II plot does show a significant increase in N_ef_ but only when measured over the past 35,000 years. The fine detail evidenced in the coding genome skyline plot likely reflects the greater phylogenetic signal in the mitochondrial coding genome relative to the HVR [33]. In general, estimates of the time to the most recent common ancestor are less sensitive to reductions in the historical signal in mtDNA sequence data than phylogenetic estimation [33], a result consistent with our ability to recover similar coalescence times but not the changes in N_ef_ seen when comparing the coding vs. HVRI+II skyline plots.

### Isolation-with-Migration Coalescent Analyses

Bayesian IM coalescent analyses were performed on a set of nine coding nuclear and mitochondrial loci that had been previously analyzed by Hey [16] in support of an extremely small New World founder N_e_ of ≈70 individuals. Thus, we performed our analysis on his identical dataset and used the same coalescent and substitution models and model parameters with the exception of new priors on the divergence time and on migration rates between Asian and Amerind populations (*m*_Asia→NW_ and *m*_NW→Asia_). The lower bound on divergence time was set to15 kya, which corresponds to the period immediately preceding the earliest archaeological evidence for human habitation in the Americas [18]–[21]. We also instituted serial constraints on *m* in order to gauge the effect of changing migration rates on founder N_e_ estimates. We interpret the various *m* values in comparison to an empirical estimate of *m* for modern Europe (*m* = 4.3; see Materials and Methods). In contrast to modern Europe, migration between the New World and Siberia from 15 kya to more recent times would have become increasingly limited as Late Pleistocene sea levels rose sufficiently to inundate the Bering land bridge [24], [25]. Thus, we expect *m* for modern Europe to be much higher than ancient migration rates between Asia and the Americas, especially after the inundation of Beringia.

**Figure 3:**
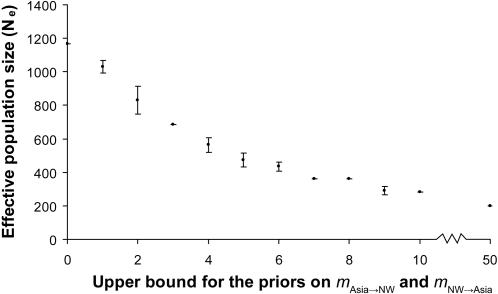
Graph of IM results for the combined nuclear and mitochondrial coding DNA dataset. The plot depicts mean N_e_ for the Amerind founder population (y-axis) as a product of increasing the constraint on the upper bound of the priors for the migration rates (x-axis). In these analyses, the prior on the lower bound of the divergence time was uniformly set to 15 kya on the basis of known archaeological materials for human occupation in the New World (see text). Each point is based on the average of the estimated medians for ten independent replicate analyses, with the bars corresponding to ± 1 standard deviation. These standard deviations are often small (with coefficients of variation less than 0.01), since their Markov chains were run for 100 million generations each.

Constraining divergence time by applying a lower bound of 15 kya results in an estimate of ≈200 for the Amerind founding N_e_. Serially constraining *m*_Asia→NW_ and *m*_NW→Asia_, in conjunction with the constrained divergence time, produces increasingly larger estimates of N_e_ (Figure 3). Specifically, as both *m* parameters are simultaneously forced to lower and more biologically realistic values, estimates of N_e_ steadily increase from ≈200 to ≈1,200, especially after their priors are constrained to be <5. Regardless of the specific priors on the *m* parameters, estimates for the Amerind divergence/expansion event are consistently ≈15 kya (data not shown), which is very close to the lower bound of our prior established with known archaeological sites in the New World. Our results demonstrate that smaller estimates of N_e_ depend upon a substantial level of migration from Asia to account for present-day levels of Amerind genetic diversity, e.g. Hey's [16] estimate of ≈70 founders is associated with a *m*_Asia→NW_>9.0, which is twice the migration rate for contemporary Europe (*m* = 4.3). Eliminating all migration between Asia and the New World (*m* = 0) results in the largest estimate of N_e_ for the Amerind founding population of ≈1,200 individuals.

## Discussion

When studying complex colonization scenarios, the interpretation of genetic data can benefit substantially from the incorporation of non-genetic material evidence. In our study, we do this in three ways. First, we interpret the skyline plot (see Figure 1) to reflect archaeological evidence that places Amerinds in the Americas by ≈15 kya and human populations in Beringia ≈30 kya, as well as geological and paleoecological evidence that Beringia was habitable yet isolated from the Americas from ≈30 kya to 17 kya. Second, we use archaeological radiocarbon dates to constrain the divergence time prior in our IM analyses to 15 kya as the latest possible date for both the divergence of the Amerind and Asian gene pools and the Amerind expansion into North America (Figure 3). Since the IM model assumes that divergence and expansion occur simultaneously, constraining the time of the expansion also requires identical constraint of the divergence date. Third, in our IM analyses we serially constrain the migration rate parameters to smaller values and deduce likely migration rates between Asia and the New World based on empirical estimates of current migration rates within Europe versus the greatly reduced migration rates of ancient people across the Bering Strait starting ≈11–10 kya.

**Figure 4:**
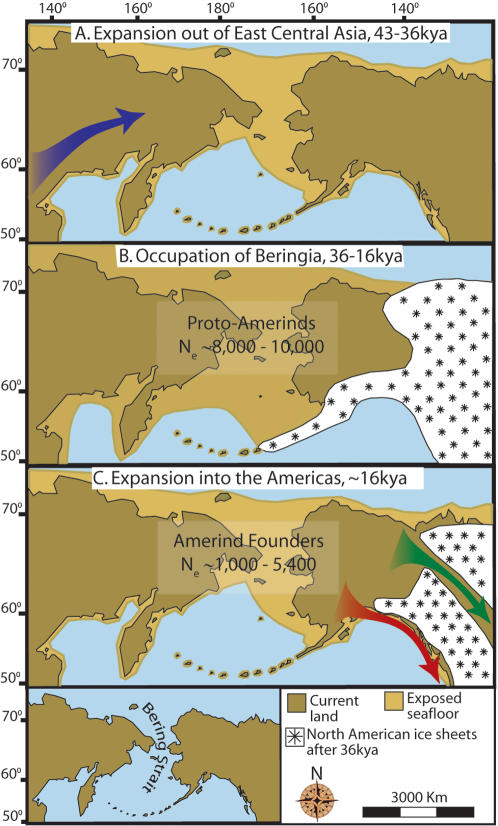
Maps depicting each phase of our three-step colonization model for the peopling of the Americas. (A) Divergence, then gradual population expansion of the Amerind ancestors from their East Central Asian gene pool (blue arrow). (B) Proto-Amerind occupation of Beringia with little to no population growth for ≈20,000 years. (C) Rapid colonization of the New World by a founder group migrating southward through the ice free, inland corridor between the eastern Laurentide and western Cordilleran Ice Sheets (green arrow) and/or along the Pacific coast (red arrow). In (B), the exposed seafloor is shown at its greatest extent during the last glacial maximum at ≈20–18 kya [25]. In (A) and (C), the exposed seafloor is depicted at ≈40 kya and ≈16 kya, when prehistoric sea levels were comparable [24], [25]. Because of the earth's curvature, the km scale (which is based on the straight line distance at the equator) provides only an approximation of the same distance between two points on these maps. In addition, a scaled-down version of Beringia today (60% reduction of A–C) is presented in the lower left corner. This smaller map highlights the Bering Strait that has geographically separated the New World from Asia since ≈11–10 kya.

Based on our results, we propose a three-stage colonization process for the peopling of the New World, with a specific focus on the dating and magnitude of the Amerind population expansions (Figure 4). We propose that the first stage was a period of gradual population growth as Amerind ancestors diverged from the central Asian gene pool and moved to the northeast. This was followed by an extended period of population stability in greater Beringia. The final stage was a single, rapid population expansion as Amerinds colonized the New World from Beringia.

The initial stage of the colonization process involved the divergence of Amerind ancestors from the East Central Asian gene pool (Figure 4A). Based on previous studies that included Asian mtDNA sequences, this divergence likely occurred prior to ≈50 kya [5], [6]. Our coding skyline plot (Figure 1) indicates that the divergence was followed by a period of gradual growth, during which the proto-Amerind population experienced a 7-fold increase from ≈640 to ≈4,400 females over ≈7,000 years, from ≈43–36 kya. The migrating founder population (N_ef_ ≈640) was a small subset of the ancestral Asian population, as evidenced by the low levels of variation in New World populations relative to Asians (e.g. [3]) as well as the larger effective size of the ancestral Asian population [16]. Thus, divergence from the Asian gene pool was the time at which a severe population bottleneck occurred that reduced the genetic variation in Amerind populations. The lack of archaeological sites in Siberia and Beringia that date to ≈43–36 kya [34] suggests that this first stage of slow population growth left a light “footprint” on the landscape because of relatively rapid and continuous movement. Consistent with this hypothesis are the younger coalescent dates for modern Siberian populations relative to modern New World populations [15], [35], which indicate that the New World migrants passed through Siberia before other East Central Asian population(s) settled permanently in this region at a later date. Such relatively rapid and continuous movement would leave few archaeological sites, which have not yet been discovered due to the vast expanse and harsh conditions of Siberia and the current inundation of Beringia. Thus, an important prediction of the first stage of our model is that older archaeological sites dating to ≈43–36 kya await discovery in these regions.

The proposed second stage (Figure 4B) consisted of an extended period of little change in population size from ≈36–16 kya (Figure 1). It is difficult to assign a precise geographic location to this population, but it may have occupied the large region from Siberia to Alaska, most of which is currently underwater. Our N_ef_ estimates of ≈4,000–5,000 (equivalent to N_e_ of ≈8,000–10,000, assuming an equal sex ratio) indicate that the proposed human presence would have been minor when compared to the size of greater Beringia. Nevertheless, the presence of this population in Beringia for ≈20,000 years would have afforded sufficient time for the generation of new mutations. Indeed, the existence of New World-specific variants that are distributed throughout the Americas indicate that substantial genetic diversification occurred during the Beringian occupation (e.g. [13]–[15], [36], [37]). The proposed period of Beringian occupation coincides with archaeological evidence for the first Arctic inhabitation of western Beringia (≈30 kya) [23] and pre-dates archaeological evidence for occupation of the New World [18]–[21]. This period also coincides with geological evidence for restricted access to North America because of the impenetrability of the Cordilleran and Laurentide ice sheets (≈17–30 kya) [38], [39]. Botanical remains, such as macrofossils and ancient pollen, indicate that Beringia was a productive grassland ecosystem rather than an exceedingly harsh Arctic desert environment [27]. Paleontological evidence from Alaska and Siberia demonstrates that large mammals such as steppe bison, mammoth, horse, lion, musk-oxen, sheep, wholly rhinoceros, and caribou inhabited this area [28].Thus, the paleoecological data are consistent with a human presence in Beringia although the carrying capacity of Beringia and technological limitations of the human population may have restricted growth until the population could expand into new and fertile lands in the Americas. The rapid expansion of the population only after an ice-free corridor into North America opened (see below) suggests that the population may have departed Beringia as soon as a viable alternative presented.

The final colonization stage (Figure 4C) was a rapid geographic expansion into the New World resulting in a significant population increase (≈16-fold; Figure 1). The rapid population increase occurred over the period ≈16–9 kya according to the coding skyline plot or over the past 15,000 years based on the IM analyses (the latter results supported only the most recent and largest expansion, most likely because IM analyses assume a single, simultaneous divergence/expansion event). The geological record indicates that North America became accessible from Beringia between ≈17–14 kya, when the ice sheets covering what is now Canada began to retreat [26], [39]. The coincident timing of an ice-free corridor into North America and the rapid expansion of the Amerind population suggests that a land route may have been the preferred entry into the New World. However, the northwest Pacific coast of North America also may have been deglaciated by ≈17 kya, thus presenting a viable coastal route to continental North America [4], [39]. This period also coincides with the initial inundation of the Bering land bridge, after which migration with Asia would have been severely limited. The first unequivocal evidence for human occupation of the New World occurs in the form of Clovis sites dating to ≈13 kya [18] and pre-Clovis sites in both North and South America dating to ≈14–15 kya [19]–[21]. Our datasets do not include typings from the Na-Dene or Esk-Aleut, so we limit our scope to the largest, initial migration of Amerinds into the New World. However, Na-Dene and Esk-Aleut genetic diversity represents a subset of Amerind diversity (e.g. [40]–[42]) suggesting that Na-Dene and Esk-Aleuts are derived from the same Beringian source population as Amerinds. As stated above, extensive archaeological evidence supports the presence of multiple distinct Native American material cultures by ≈13 kya (e.g. Clovis, Nenana and pre-Clovis lithic technologies [18]). Our results suggest that these distinct cultures derive from a single New World founder population and are most likely the product of an extensive and complex process of post-peopling migrations within the Americas, possibly through a combination of coastal and/or riverine routes [4], [43].

Determination of the size of the Amerind founding population has received considerable attention. Based on the coding Bayesian skyline plot (Figure 1), there is a slight decrease in population size preceding the increase seen at ≈15 kya. This decrease is consistent with a secondary founder effect in which a subset of the Beringian population seeded the proto-Amerind expansion into the Americas. Assuming the apparent decrease in N_ef_ is the result of such a founder effect, the upper bound on the founder population size is ≈5,400 individuals (N_ef_ ≈2,700). Our IM analyses suggest that the founder population size could be lower depending on prior assumptions about the over water migration rates between the Americas and Asia (see Figure 3). Migration rates (*m*) within Europe today based on census data have been determined to be 4.3, which can be taken as an extreme upper bound of possible ancient migration rates between the Americas and Asia, especially after the appearance of the Bering Strait ≈11–10 kya. Restricting migration rates to <1 results in founder N_e_ estimates between ≈1,000 and ≈1,200, with ≈1,200 serving as an asymptotic upper bound (see Figure 3). Taken together, our Bayesian skyline plot and IM analyses suggest that a founder population with N_e_ = 1,000–5,400 colonized the New World in a process characterized by a rapid geographic and population expansion. The range of N_e_ values can be translated into an approximate census population size by applying a scale factor estimated from large mammal populations (scale factor = 5) [44], which suggests that the founder population consisted of ≈5,000–27,000 people.

Our three-stage model now awaits further critical testing with new datasets of independent nuclear loci and more sophisticated methods of coalescent analysis. The extensive dataset of ≈700 autosomal microsatellites, compiled by Wang et al. [4] for both Native American and worldwide populations, offers the opportunity to evaluate critically the size, timing, and duration of each step in our model at essentially a population genomics level. Future versions of BEAST will incorporate a structured coalescent where migration as well as population growth will be allowed to occur among populations from both the New World and Asia (http://evolve.zoo.ox.ac.uk/beast/manual.html). In these BEAST analyses, the microsatellites can be modeled under a stepwise “ladder process,” whereby alleles are inter-related according to their repeat lengths. One can then summarize over these microsatellite loci by assuming independence, which thereby allows for the multiplication of their separate posterior distributions and final estimations of their combined Bayesian skyline plot. In these ways, we fully anticipate that such critical testing will lead to many important refinements of our three-step model, including a further narrowing of our proposed range for the size of the founding population as well as new details about post-peopling expansions within the New World.

## Materials and Methods

### Datasets

Three datasets were collected for analysis including: (*i*) 77 mtDNA coding genomes; (*ii*) 812 mtDNA HVRI+II sequences; and (*iii*) combined nuclear and mitochondrial coding DNA dataset. The 77 mtDNA coding genomes were collected from publicly available resources [45]–[48] and aligned using ClustalX [49]. The resultant 15,500 base pair (bp) multiple alignment was edited by hand to minimize the number of unique gaps and to ensure the integrity of the reading frame (available online as Figure S1). A total of 812 combined HVRI+II sequences were collected from HVRbase (http://www.hvrbase.org) [50]. These sequences were aligned following the coding mtDNAs, resulting in a multiple alignment of 771 bps (available online as Supplemental Figure S2). The complete dataset of 812 HVRI+II sequences was randomly divided into ten non-overlapping alignments of 81 sequences that approximate the sample size for the coding mtDNA dataset. Skyline plot analyses of larger datasets (up to 200 HVRI+II sequences) gave the same results as the 81 sequence datasets (data not shown). Thus, the smaller datasets of 81 sequences each were emphasized here since they avoided the likelihood rounding errors that can occur when using large, heterogeneous datasets in Bayesian skyline plot analyses. The coding nuclear and mtDNA dataset from Asian and Native American populations of Hey (available at http://lifesci.rutgers.edu/heylab/) [16] consisted of two autosomal coding loci, five X-chromosome coding loci, one Y-chromosome coding locus, and the complete mtDNA coding genome (totaling 28,454 aligned bps). The sample sizes for these nuclear loci and mitochondrial genome varied from 12-108 sequences.

### Bayesian Skyline Plot Analyses

Bayesian skyline plots [32] were used to estimate changes in Amerind N_ef_ over time by providing highly parametric, piecewise estimates of N_ef_. This approach produces serial estimates of effective population size from the time intervals between coalescent events in a genealogy of sampled individuals, and utilizes a Markov chain Monte Carlo simulation approach to integrate over all credible genealogies and other model parameters. It thereby differs from previous approaches (e.g., [51]) in that Bayesian skyline plots fully parameterize both the mutation model (including relaxed clock models) and the genealogical process, whereas prior methods relied on generating estimates from summary statistics (e.g. the use of pairwise differences by [51]).

In these analyses, estimates of τ (N_ef_×generation time) were converted to N_ef_ by dividing by a generation time of 20 years, following convention [16]. Skyline plots were generated for the 77 mtDNA coding genome sequences and the ten datasets of HVRI+II sequences using the program BEAST v1.4 (http://beast.bio.ed.ac.uk). These BEAST analyses relied on the same coalescent and substitution models and run conditions of Kitchen *et al.* [52] except as noted below. Plots were generated using the established mutation rates (μ) for coding mtDNA (μ = 1.7×10^−8^ substitutions/site/year) [46] and HVRI+II mtDNA (μ = 4.7×10^−7^) [53]. Markov chains were run for 100,000,000 generations and sampled every 2,500 generations with the first 10,000,000 generations discarded as burn-in. Three independent runs were performed for all coding and HVRI+II Bayesian skyline plot analyses. Markov chain samples from the three independent mtDNA coding replicates and from the 30 HVRI+II analyses were separately combined using the LogCompiler program (distributed with BEAST) and analyzed using Tracer v1.3 to produce the final Bayesian skyline plots.

### Isolation-with-Migration Coalescent Analyses

Bayesian IM coalescent analyses were performed using the program IM [16] to estimate N_e_ for the Amerind founder population (males+females) and the divergence time for Amerind and Asian populations. We used the same combined nuclear and mtDNA dataset, same coalescent and substitution models, and same model parameters as Hey [16] with the exception of new priors on the divergence time and on the migration rates between Asian and Amerind populations. All IM analyses were performed using a flat uniform prior for the divergence time of Amerind and Asian populations set to the interval 15–40 kya. The lower bound of this prior is based on accepted archaeological and climatological evidence for the first presence of humans in the Americas [18]–[21]. The upper bound of the flat uniform priors on the migration rates per mutation per generation between the Amerindian and Asian populations (*m*_Asia→NW_ and *m*_NW→Asia_) was set to 12 different values (0, 1, 2, 3, 4, 5, 6, 7, 8, 9, 10, and 50). To help interpret these results, we relied on an estimate of the migration rate in modern Europe as obtained from census data [54]. Specifically, we converted their migration rate estimate of 0.0004 migrations per gene copy per generation (recalculated assuming a generation time of 20 years based on Hey [16]) to our units of migrations per mutation per generation (*m*) by dividing the former by the geometric mean of the mutation rates for the nine loci in this dataset (9.32×10^−5^ mutations per locus per generation ). These calculations resulted in *m* = 4.3 for modern Europe. In contrast, the ancient migration rates between the New World and Asia would have been significantly less, especially after their geographic separation due to the re-inundation of Beringia starting at ≈11 kya (see Introduction). Ten independent replicates were performed for each of the 12 upper bound values on the migration rates, for a total of 120 IM analyses. All Markov chains were run for 100,000,000 generations without heating.

## Supporting Information

Figure S1 Multiple sequence alignment for the 77 Amerind mtDNA coding genomes used in this study. Here, “coding” refers to both protein and structural RNA genes following Pakendorf and Stoneking [30]. Gaps are represented by “-.” Position 1 of this alignment corresponds to site 546 of the Anderson Reference Sequence (ARS; [56]). The final position of this alignment (15,500) corresponds to site 16,042 of the ARS. Sequences starting with “Herrn,” “Ing,” “Kiv”, and “Mis” follow the naming conventions of Herrnstadt et al. [45], Ingman et al. [46], Kivisild et al. [47], and Mishmar et al. [48], respectively.
(1.19 MB TXT)

Figure S2 Multiple sequence alignments for the ten, randomly selected, non-overlapping sets of 81 HVRI+II sequences used in this study. In these alignments, positions 1-403 correspond to HVRI, whereas sites 404-781 refer to HVRII. In turn, these alignment positions correspond to sites 16003-16400 and 30-399 of the ARS, respectively. Gaps are represented by “-.” The HVRI+II sequences follow the naming conventions of HRVBase [50].
(0.64 MB TXT)
